# An Indole-Based Fluorescent Chemosensor for Detecting Zn^2+^ in Aqueous Media and Zebrafish

**DOI:** 10.3390/s21165591

**Published:** 2021-08-19

**Authors:** Donghwan Choe, Haeri So, Soyoung Park, Hangyul Lee, Ju Byeong Chae, Jiwon Kim, Ki-Tae Kim, Cheal Kim

**Affiliations:** 1 Department of Fine Chem and Renewable Energy Convergence, Seoul National University of Science and Technology (SNUT), Seoul 139-743, Korea; ehdghksdl_@naver.com (D.C.); gofl0988@naver.com (H.S.); soyp19@gmail.com (S.P.); bonbongyul@gmail.com (H.L.); ch920812@naver.com (J.B.C.); 2 Department of Environmental Engineering, Seoul National University of Science and Technology (SNUT), Seoul 139-743, Korea; jiwonss6408@naver.com

**Keywords:** zinc ion, indole, fluorescent chemosensor, calculations, bio-imaging

## Abstract

An indole-based fluorescent chemosensor **IH-Sal** was synthesized to detect Zn^2+^. **IH-Sal** displayed a marked fluorescence increment with Zn^2+^. The detection limit (0.41 μM) of **IH-Sal** for Zn^2+^ was greatly below that suggested by the World Health Organization. **IH-Sal** can quantify Zn^2+^ in real water samples. More significantly, **IH-Sal** could determine and depict the presence of Zn^2+^ in zebrafish. The detecting mechanism of **IH-Sal** toward Zn^2+^ was illustrated by fluorescence and UV–visible spectroscopy, DFT calculations, ^1^H NMR titration and ESI mass.

## 1. Introduction

Zinc ion, the second richest in body, has essential roles related to various physiological functions like gene transcription and immune and brain functions [1,2,3,4,5,6,7,8]. However, the imbalance of zinc ions may result in several pathological problems, such as epilepsy, infantile diarrhea, Parkinson’s disease, ischemic stroke and Alzheimer’s disease [9,10,11]. Thus, effective probing and monitoring of zinc ions in biological systems has become an important issue [12].

Various analytical methods, like electrochemical methods, inductively coupled plasma atomic emission spectroscopy (ICP-AES) and atomic absorption spectrometry (AAS), have been applied for determining zinc ions [13,14]. However, they require complicated sample preparation, expensive instruments and time-consuming procedures [15]. By contrast, fluorescent chemosensors have merits such as high selectivity, simplicity and low cost [16,17,18,19,20,21,22]. Moreover, fluorescent chemosensors could be applied to living organisms for bio-imaging [23,24,25,26,27]. Meanwhile, it is a huge obstacle to distinguish zinc ions from cadmium ions, since they show similarity in chemical properties [28,29,30]. Thus, chemosensors capable of discriminating zinc ions from cadmium ions are especially needed.

Indole derivatives have been widely applied to chemosensors for detecting various ions, such as F^−^, CN^−^, I^−^, Cu^2+^ and Hg^2+^ [31,32,33,34,35], because of their unique fluorescent characters and good water solubility [36,37]. In addition, they are bio-compatible and essential in biological systems [38,39,40,41]. As a result, some of the indole-based chemosensors have shown applications in aqueous media, which contributed to bio-imaging [42,43,44,45]. Nevertheless, only five indole-based Zn^2+^ chemosensors have been reported to date, and only one of them presented an application in living organisms [46,47,48,49,50].

Herein, we demonstrate an indole-based fluorescent probe **IH-Sal** for probing Zn^2+^, that was provided by condensation reaction of 2-(1H-indol-3-yl)acetohydrazide and salicylaldehyde. **IH-Sal** showed efficient fluorescence turn-on for Zn^2+^ and could be applied to recognize and quantify Zn^2+^ in real samples and zebrafish.

## 2. Experiments

### 2.1. Materials and Equipment

Reagents were provided commercially. Electrospray ionisation mass spectrometry (ESI-MS) and nuclear magnetic resonance spectroscopy (NMR) data were provided with a Thermo Finnigan quadrupole instrument (Thermo Finnigan LLC, San Jose, CA, USA) and a Varian spectrometer (Varian, Palo Alto, CA, USA). Fluorescent and UV–visible spectra were provided by Perkin Elmer spectrometers (Perkin Elmer, Waltham, MA, USA).

### 2.2. Synthesis of IH-Sal ((E)-N′-(2-hydroxybenzylidene)-2-(1H-indol-3-yl)acetohydrazide)

**Scheme 1:**
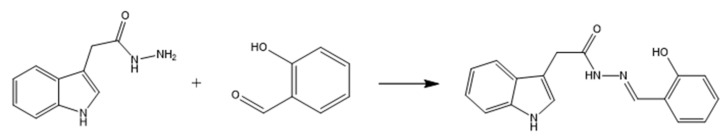
Synthesis of **IH-Sal** (see the experimental section for details).

Following the method for synthesizing **IH-Sal** reported in the literature [51], salicylaldehyde (61.1 mg, 5 × 10^−4^ mol) was added to 2-(1H-indol-3-yl)acetohydrazide (100.3 mg, 5.3 × 10^−4^ mol) in methanol (2 mL) with stirring for 2 h at 23 °C (Scheme 1). A white precipitate was filtered, rinsed with methanol and dried (118.1 mg; 80.5%); ^1^H NMR in DMSO-*d_6_*: δ 11.77 (s, 0.67H), 11.27 (s, 0.33H), 11.16 (s, 0.67H), 10.94 (s, 0.67H), 10.88 (s, 0.33H), 10.12 (s, 0.33H), 8.41 (s, 0.67H), 8.28 (s, 0.33H), 7.72–6.86 (m, 9H), 4.01 (s, 0.67H), 3.65 (s, 1.33H). ^13^C NMR in DMSO-*d_6_*: δ 172.1 (0.33C), 166.9 (0.67C), 157.2 (0.67C), 156.2 (0.33C), 146.7 (0.67C), 136.0 (0.33C), 131.1 (1C), 130.8 (1C), 129.3 (1C), 127.0 (1C), 123.8 (1C), 123.7 (1C), 121.0 (1C), 120.7 (1C), 118.5 (1C), 118.3 (1C), 116.2 (1C), 111.4 (1C), 107.7 (1C), 31.3 (0.67C), 29.2 (0.33C). ESI-MS (*m*/*z*): [**IH-Sal** + H^+^ + DMSO]^+^: calculated, 372.14, found, 372.25.

### 2.3. Preparation of Spectroscopic Experiments

Sensor **IH-Sal** (2.93 mg, 10 μmol) was dissolved in DMSO (1 mL) for a stock solution (10 mM). A Zn^2+^ stock (20 mM) was prepared by dissolving Zn(NO_3_)_2_ in bis-tris buffer (1 × 10^−2^ M, pH 7). We also prepared other metal ion stocks using their nitrate salts or perchlorate salts, such as Ga(NO_3_)_3_, Co(NO_3_)_2_, NaNO_3_, Cr(NO_3_)_3_, Fe(ClO_4_)_2_, Ca(NO_3_)_2_, Fe(NO_3_)_3_, Pb(NO_3_)_2_, Mn(NO_3_)_2_, Ni(NO_3_)_2_, Cd(NO_3_)_2_, Mg(NO_3_)_2_, In(NO_3_)_3_, Cu(NO_3_)_2_, Al(NO_3_)_3_ and KNO_3_. All spectroscopic experiments were conducted immediately after mixing them for a few seconds.

### 2.4. Imaging in Zebrafish

Zebrafish embryos were reared under previously described conditions [52,53]. The 6-day-old embryos were treated with 2 × 10^−5^ M of **IH-Sal** (containing 0.02% DMSO in E2 media) for 21 min. After washing with E2 media to eliminate the remnant **IH-Sal**, the embryos were treated with two different amounts of Zn^2+^ solution (2.5 and 5.0 × 10^−5^ M) in E2 media for 20 min and washed again. Before observing changes, the embryos were narcotized by adding ethyl-3-aminobenzoate methanesulfonate. An imaging experiment was performed with a fluorescent microscope and the intensity of the images was measured by Icy software (Institut Pasteur, Paris, France).

### 2.5. Calculations

The results of theoretical calculations were given with the Gaussian 16 program (Gaussian, Inc., Wallingford, CT, USA) [54]. Before calculating electronic states of **IH-Sal** and **IH-Sal**-Zn^2+^ complex, their optimal geometries were provided with the density functional theory (DFT) method [55,56]. The hybrid functional was B3LYP, and the 6-31G(d,p) basis set was implemented for all atoms except for Zn^2+^ [57,58]. Additionally, the LANL2DZ basis set was applied for effective core potentials (ECP) to Zn^2+^ [59,60,61]. Imaginary frequency was not shown in optimized forms of **IH-Sal** and **IH-Sal**-Zn^2+^, implying that they meant local minima. With IEFPCM, the solvent effect of water was considered [62]. Based on energy-optimized forms of **IH-Sal** and the **IH-Sal**-Zn^2+^ complex, the plausible UV–Vis transition states were verified with the DFT method with the twenty lowest singlet states.

## 3. Results and Discussion

### 3.1. Structural Characterization of IH-Sal

The ^1^H NMR of **IH-Sal** showed pairs of singlets having a 1:2 ratio of integral value for the protons H_1_, H_6_, H_8_, H_9_ and H_14_, implying that it has two isomeric forms originated from keto-enol tautomerization (Figure S1). The compound **IH-Sal** was further verified by ^13^C NMR and ESI-MS.

### 3.2. Spectroscopic Examination of IH-Sal to Zn^2+^

**Figure 1:**
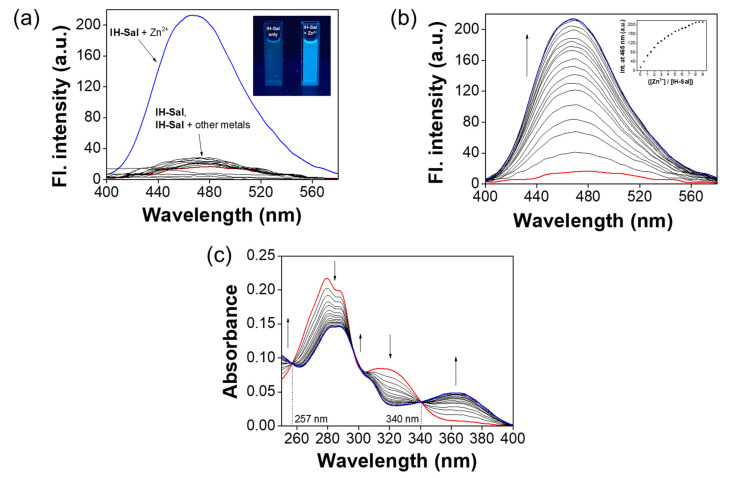
(**a**) Fluorescence changes in **IH-Sal** (1 × 10^−5^ M) with varied cations (8.5 equiv). Photograph: the fluorescence images of **IH-Sal** and **IH-Sal**-Zn^2+^ under UV light (λ_ex_: 369 nm); (**b**) fluorescence titration of **IH-Sal** (1 × 10^−5^ M) with varied amounts of Zn^2+^ (0–9 equiv); (**c**) UV–Visible variations in **IH-Sal** (1 × 10^−5^ M) with varied amounts of Zn^2+^ (0–8 equiv).

To comprehend the fluorescent characteristic of **IH-Sal**, the fluorescent variation was checked with varied cations in bis-tris buffer (Figure 1a). **IH-Sal** itself exhibited no fluorescence emission. Upon the addition of the cations except for Zn^2+^, **IH-Sal** displayed either no variation or a trivial increase in the fluorescent emissions. Meanwhile, the addition of Zn^2+^ displayed a striking fluorescence increment at 465 nm (λ_ex_ = 369 nm) with a large stokes shift. The stokes shift was the largest among indole-based Zn^2+^ sensors (Table S1). The quantum yields (*Φ*) of **IH-Sal** and **IH-Sal**-Zn^2+^ were calculated to be 0.014 and 0.153, respectively. Therefore, **IH-Sal** can work as a fluorescence sensor for a clearly selective probing of Zn^2+^. In the literature, the displacement of the indole moiety in **IH-Sal** by a benzene ring or tetraphenylethylene showed that the sensors sensed Zn^2+^ ions only in organic or semi-aqueous solvents [63,64], confirming that the indole moiety might play an important role in increasing water solubility of **IH-Sal**.

To demonstrate the sensing characteristics of **IH-Sal** to Zn^2+^, a fluorescence titration of **IH-Sal** and Zn^2+^ was conducted (Figure 1b). The fluorescence intensity of **IH-Sal** at 465 nm consistently increased up to 8.5 equivalent (equiv) of Zn^2+^. The photophysical characteristics of **IH-Sal** were also tested with UV–Vis spectrometry (Figure 1c). With the addition of Zn^2+^ to **IH-Sal**, the absorption of 250 and 360 nm consistently increased, and that of 290 and 320 nm decreased. There were clean isosbestic points at 257 and 340 nm, implying that one species was provided by the complexation of **IH-Sal** with Zn^2+^. On the other hand, the UV–Vis change in **IH-Sal** with various metal ions showed that **IH-Sal** was not selective to Zn^2+^ (Figure S2).

To confirm the stoichiometry of complexation, the Job plot experiment was carried out (Figure S3). The biggest intensity was shown at a mole fraction of 0.5, suggesting that **IH-Sal** and Zn^2+^ formed a 1:1 binding compound. The 1:1 binding of **IH-Sal**-Zn^2+^ was verified by ESI-MS analysis (Figure S4). Positive ion mass displayed that the peak of 511.58 (*m*/*z*) was suggestive of [**IH-Sal**(-H^+^) + Zn^2+^ + 2DMSO]^+^ (calculated, 512.07). Based on the stoichiometry, the Benesi–Hildebrand equation [65,66] was used to calculate *K* (association constant) for **IH-Sal**-Zn^2+^ (Figure S5). The *K* value was given to be 1.6 × 10^4^ M^−1^, which was within the scope of those (1~1.0 × 10^13^) addressed for Zn^2+^ sensors.

**Figure 2:**
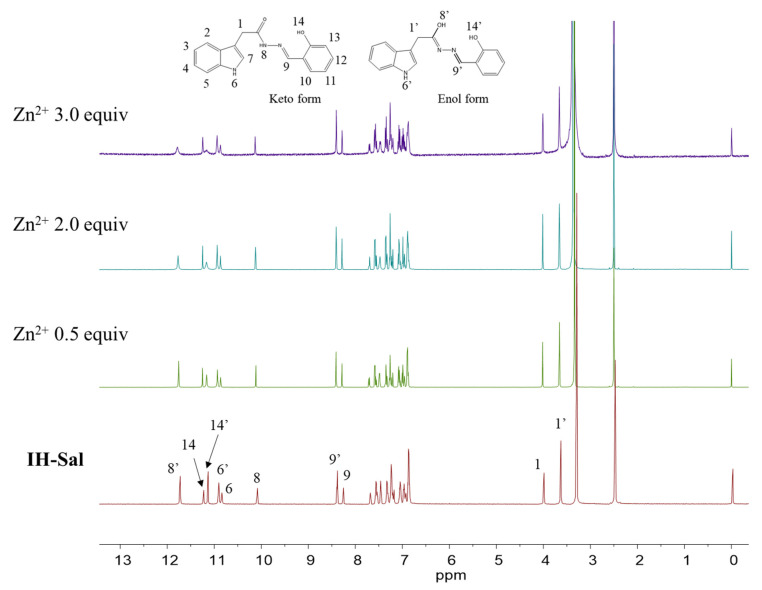
^1^H NMR titration of **IH-Sal** with Zn^2+^ (0, 0.5, 2.0 and 3.0 equiv).

**Scheme 2:**
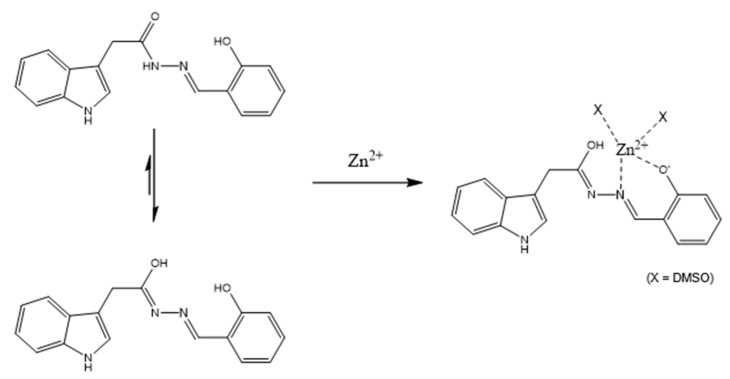
Appropriate structure of **IH-Sal**-Zn^2+^.

The ^1^H NMR titrations were executed to demonstrate the binding interaction of **IH-Sal** and Zn^2+^ (Figure 2). With the addition of Zn^2+^ to **IH-Sal**, the proton H_14′_ disappeared and the proton H_9_ was slightly moved to upfield. These results implied that the enol form of **IH-Sal** could interact with Zn^2+^ using the oxygen of the deprotonated phenol and the nitrogen of the imine group (Scheme 2).

We used **IH-Sal** to measure the amount of Zn^2+^ in real water samples, based on a calibration plot of **IH-Sal** to Zn^2+^ (Figure S6). As real water samples, we chose tap and drinking water (Table 1). Quantification of each sample was repeated twice and showed proper recovery and relative standard deviation (R.S.D.), indicating that **IH-Sal** could work as an efficient chemosensor for monitoring Zn^2+^ in real samples. From the calibration curve, the detection limit of **IH-Sal** for zinc ions was calculated to be 0.41 μM based on 3σ/k, which was greatly below that suggested by the WHO (76.0 μM) for Zn^2+^ ions [67]. The value is the lowest among those previously found for indole-based Zn^2+^ chemosensors in a near-perfect aqueous solution (Table S1).

To prove the practicability of **IH-Sal** as a practical probe for zinc ions, competitive tests were executed (Figure S7). With the same amount of Zn^2+^ and other cations with **IH-Sal**, most cations did not inhibit the sensing ability of **IH-Sal** for zinc ions. However, Cu^2+^, Fe^2+^, Cr^3+^, Fe^3+^ and Co^2+^ interfered with the fluorescence emission of **IH-Sal** with Zn^2+^.

**Figure 3:**
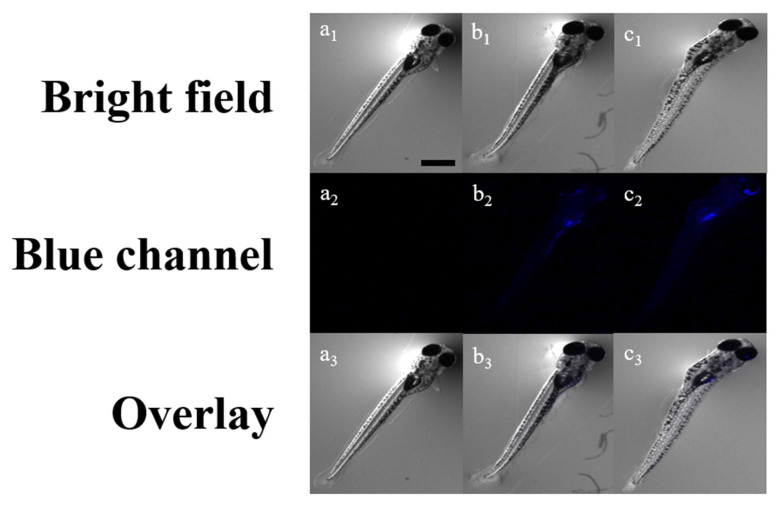
Fluorescent images of zebrafish cultured with **IH-Sal** followed by addition of Zn^2+^. (**a_1_**–**a_3_**): **IH-Sal** only; (**b_1_**–**b_3_**): **IH-Sal** with 2.5 × 10^−5^ M Zn^2+^; (**c_1_**–**c_3_**): **IH-Sal** with 5 × 10^−5^ M Zn^2+^. [**IH-Sal**] = 2.0 × 10^−5^ M. Scale bar: 0.91 mm.

The pH dependence of **IH-Sal**-Zn^2+^ for biological application was tested with various pH values (6–9, Figure S8). While there was no fluorescence emission at pH 6, **IH-Sal**-Zn^2+^ showed a remarkable fluorescence response between pH 7 and 9, indicating that **IH-Sal** can clearly recognize Zn^2+^ by the fluorescence application within the environmental pH range [68]. Based on the result of the pH dependence, fluorescence imaging of zebrafish was performed to widen the biological application. While the zebrafish cultured with **IH-Sal** (20 μM) alone did not show any fluorescent signal (Figure 3), blue emission on the zebrafish cultured with **IH-Sal** gradually increased as the amount of Zn^2+^ increased from 0 to 50 μM. The mean intensity of the images was calculated with Icy software (Figure S9), given the detection limit of 5.07 μM. These results supported the biocompatibility of **IH-Sal** as a useful fluorescent probe for sensing Zn^2+^ in live organisms. Importantly, this is the second indole-based Zn^2+^ chemosensor for application to living organisms (Table S1).

### 3.3. Calculations

**Figure 4:**
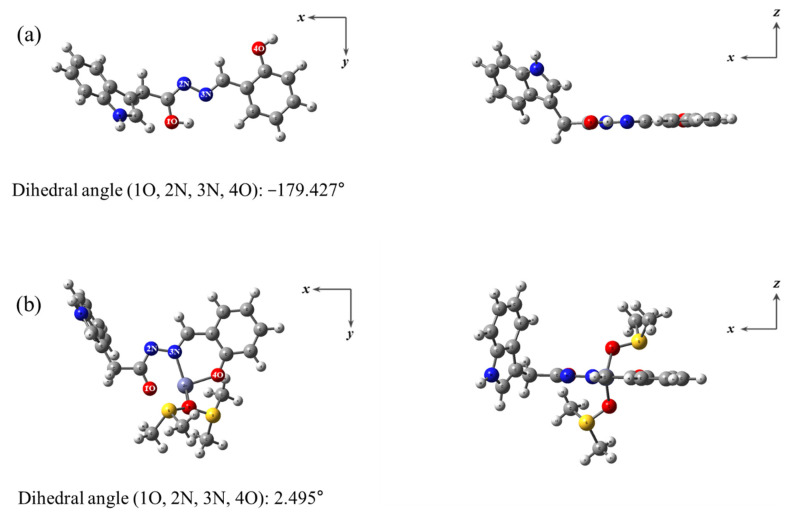
Energy-optimized patterns of (**a**) **IH-Sal** and (**b**) **IH-Sal**-Zn^2+^.

With the results of the Job plot and ESI mass, the optimal structures of **IH-Sal**-Zn^2+^ and **IH-Sal** were provided with DFT calculation (Figure 4). **IH-Sal** with a dihedral angle of −179.427° (1O, 2N, 3N, 4O) had a moderately distorted structure (Figure 4a). **IH-Sal**-Zn^2+^ had a structure with a flipped phenol group (Figure 4b), showing a dihedral angle of 2.495°. Based on the energy-optimized forms of **IH-Sal** and **IH-Sal**-Zn^2+^, transition energies and molecular orbitals were examined with TD-DFT calculations. For **IH-Sal**, the main absorption of HOMO-1→LUMO transition (314.18 nm) exhibited π→π* transition (Figure S10). The major absorption of **IH-Sal**-Zn^2+^ derived from HOMO→LUMO transition (358.71 nm, Figure S11) also displayed π→π* transition (Figure S12). The red shift (320 to 360 nm) shown in the UV–Vis spectra was greatly matched with the calculated transitions and corresponded to a decreased energy gap. These outcomes implied that the fluorescence turn-on of **IH-Sal** to Zn^2+^ may be a chelation-enhanced fluorescence (CHEF) effect [69]. With the Job plot, ESI-MS, ^1^H NMR titration and calculations, the appropriate structure of **IH-Sal**-Zn^2+^ is proposed in Scheme 2.

## 4. Conclusions

We illustrated an indole-based fluorescent probe, **IH-Sal**, which was produced from the condensation of 2-(1H-indol-3-yl)acetohydrazide and salicylaldehyde. **IH-Sal** could work as an effective fluorescent probe for monitoring Zn^2+^. The detection limit (0.41 μM) for Zn^2+^ was significantly below that suggested by the WHO (76.0 μM). The value is the lowest among those previously found for indole-based Zn^2+^ chemosensors in a near-perfect aqueous solution. **IH-Sal** could be reliably applied to real samples and showed its practical applicability to recognize Zn^2+^ in zebrafish. Importantly, this is the second indole-based Zn^2+^ chemosensor for application to living organisms. Thus, we believe that **IH-Sal** can be an efficient fluorescent chemosensor to determine Zn^2+^ in biological and practical applications.

## Figures and Tables

**Table 1:** Determination of Zn^2+^. ^a^

Sample	Zn^2+^ Added (μM)	Zn^2+^ Found (μM)	Recovery (%)	R.S.D. (*n* = 3) (%)
Drinking water	0.0	0.0	-	-
	10.0	10.0	100.01	1.58
Tap water	0.0	0.0	-	-
	10.0	10.1	101.00	0.40

^a^ Conditions: [**IH-Sal**] = 1 × 10^−5^ M in buffer.

## Data Availability

Not applicable.

## Supplementary Materials

The following are available online at https://www.mdpi.com/article/10.3390/s21165591/s1, Table S1: Examples of indole-based Zn^2+^ chemosensors found to date; Figure S1: 1H NMR spectrum of **IH-Sal**; Figure S2: UV–Vis changes in **IH-Sal** (1 × 10^−5^ M) with various metal ions (8 equiv); Figure S3: Job plot for the binding of **IH-Sal** with Zn^2+^ (50 μM) in bis-tris buffer (10 mM, pH 7.0); Figure S4: Positive-ion ESI mass spectrum of **IH-Sal** (100 μM) upon the addition of 1 equiv of Zn^2+^; Figure S5: Benesi–Hildebrand equation plot (at 465 nm) of **IH-Sal** (10 μM) based on fluorescence titration, assuming 1:1 stoichiometry for association between **IH-Sal** and Zn^2+^; Figure S6: Calibration curve of **IH-Sal** as a function of Zn^2+^ concentration; Figure S7: Competitive selectivity of **IH-Sal** (10 μM) toward Zn^2+^ (8.5 equiv) in the presence of other metal ions (8.5 equiv, λ_ex_ = 369 nm); Figure S8: Fluorescent intensity of **IH-Sal** (10 μM) and **IH-Sal**-Zn^2+^ species, respectively, at different pH values (6–9); Figure S9: Quantification of mean fluorescence intensity in Figure S7 (a_2_, b_2_ and c_2_); Figure S10: (a) The theoretical excitation energies and the experimental UV–Vis spectrum of **IH-Sal**. (b) The major electronic transition energies and molecular orbital contributions of **IH-Sal**; Figure S11: (a) The theoretical excitation energies and the experimental UV–Vis spectrum of **IH-Sal**-Zn^2+^. (b) The major electronic transition energies and molecular orbital contributions of **IH-Sal**-Zn^2+^; Figure S12: The major molecular orbital transitions and excitation energies of **IH-Sal** and **IH-Sal**-Zn^2+^.
