# Supplementary material for: An Indole-Based Fluorescent Chemosensor for Detecting Zn2+ in Aqueous Media and Zebrafish
Source: Sensors (Basel). 2021 Aug 19;21(16):5591. doi: 10.3390/s21165591 (PMC8402251; doi:10.3390/s21165591)
Supplement: Supplementary file 1 [file sensors-21-05591-s001.zip › sensors-1327105-supplementary.pdf]

Supplementary Materials

An Indole-Based Fluorescent Chemosensor for Detecting Zn^{2+} in Aqueous Media and Zebrafish

Donghwan Choe [1], Haeri So [1], Soyoung Park [1], Hangyul Lee [1], Ju Byeong Chae [1], Jiwon Kim [2], Ki-Tae Kim [2,*] and Cheal Kim [1,*]

[1] Department of Fine Chem and Renewable Energy Convergence, Seoul National University of Science and Technology (SNUT), Seoul 139-743, Korea; ehdghksdl_@naver.com (D.C.); gofl0988@naver.com (H.S.); soyp19@gmail.com (S.P.); bonbongyul@gmail.com (H.L.); ch920812@naver.com (J.B.C.)

[2] Department of Environmental Engineering, Seoul National University of Science and Technology (SNUT), Seoul 139-743, Korea; jiwonss6408@naver.com

[*] Correspondence: ktkim@seoultech.ac.kr (K.-T.K.); chealkim@snut.ac.kr (C.K.); Tel.: +82-2-962-6642 (K.-T.K.); +82-2-972-6673 (C.K.); Fax: +82-2-981-9147 (C.K.)

Table S1. Examples of indole-based Zn^{2+} chemosensors found to date.

No.	Structure	Detection limit (μM)	Application to living organism (detection limit (μM))	Stokes shift (nm)	Solvent	Reference
1	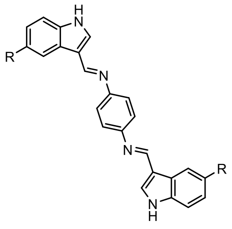 (R = H, OCH_3)	0.1	No	−28	DMF	[1]
2	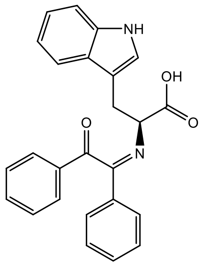	1	No	55	CH_3OH : H_2O (v/v = 1:1)	[2]
3	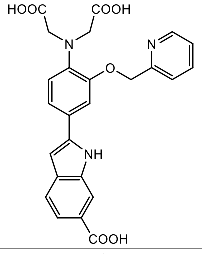	None	No	60	HEPES buffer (50 mM, pH 7.2)	[3]
4	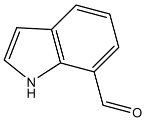	None	No	80	Water	[4]
5	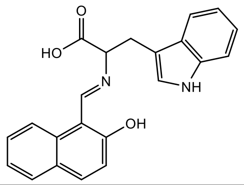	None	Yes (None)	26	Tris-HCl buffer (10 mM, pH 7.5)	[5]
6	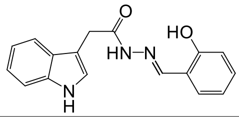	0.41	Yes (5.07)	96	Bis-tris buffer (10 mM, pH 7.0)	This work

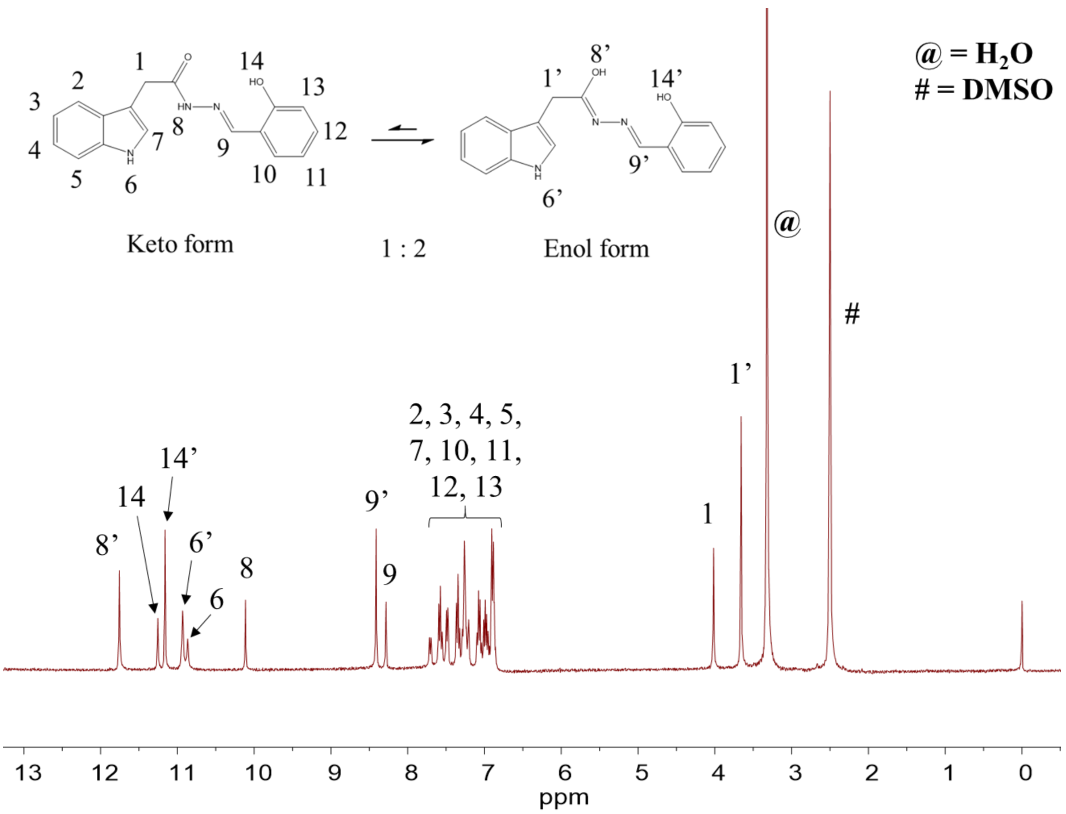

Figure S1. ^{1}H NMR spectrum of **IH-Sal**.

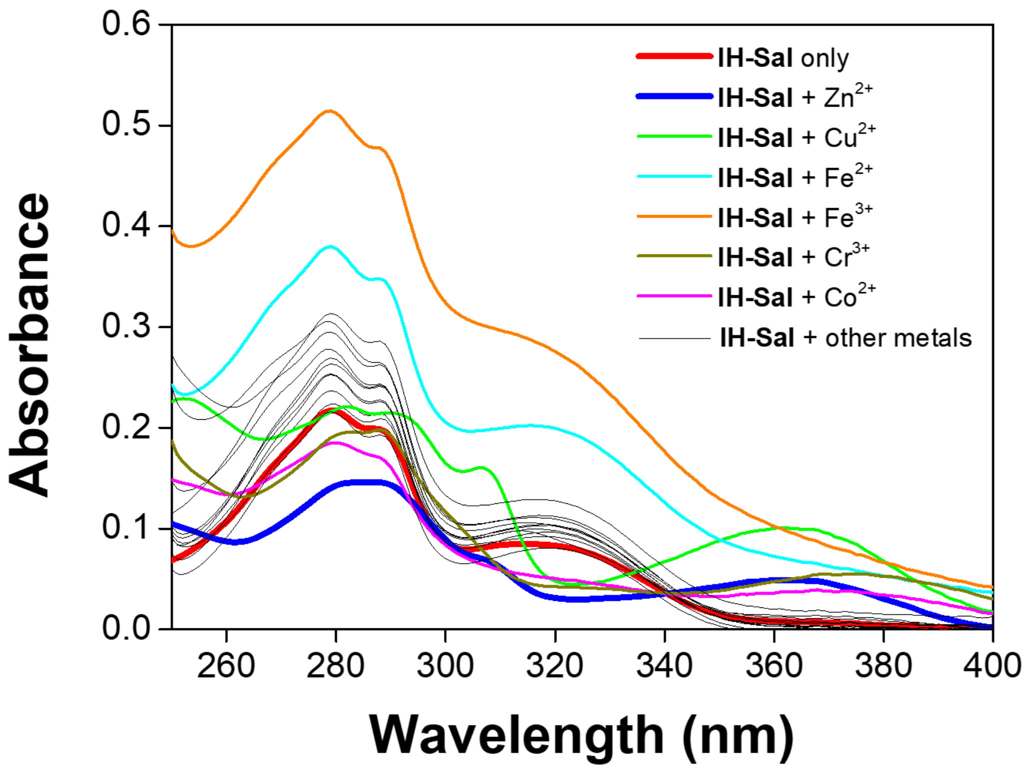

Figure S2. UV–Vis changes in **IH-Sal** (1×10^{-5} M) with various metal ions (8 equiv).

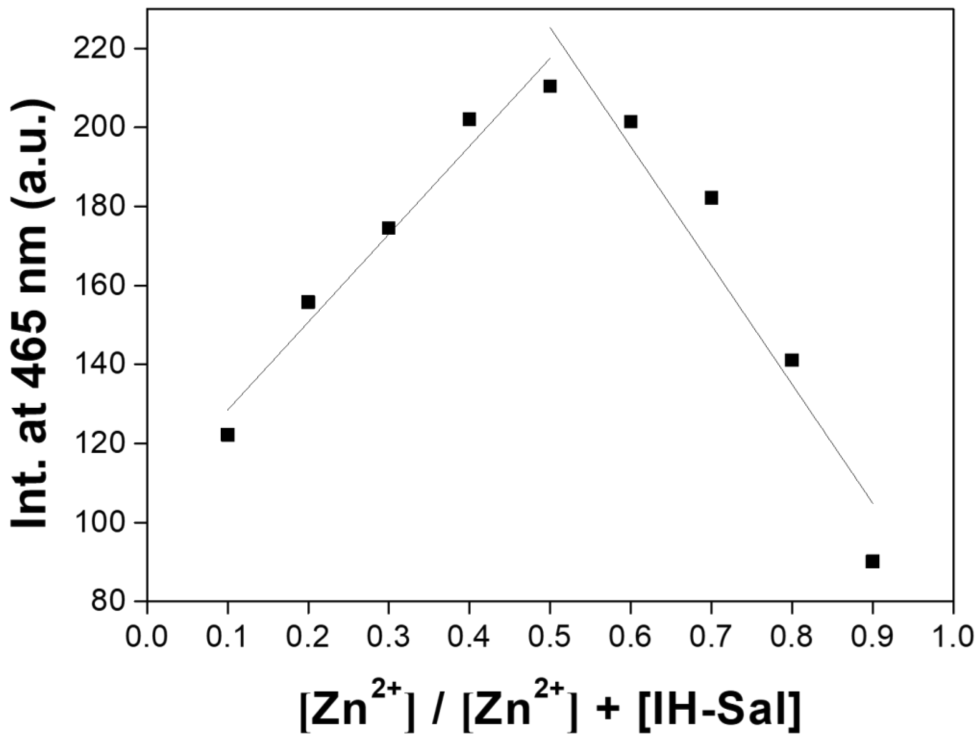

Figure S3. Job plot for the binding of **IH-Sal** with Zn^{2+} (50 μM) in bis-tris buffer (10 mM, pH 7.0). Fluorescence intensity at 465 nm was plotted as a function of the molar ratio of $[Zn^{2+}]/([Zn^{2+}]+[$**IH-Sal**$])$.

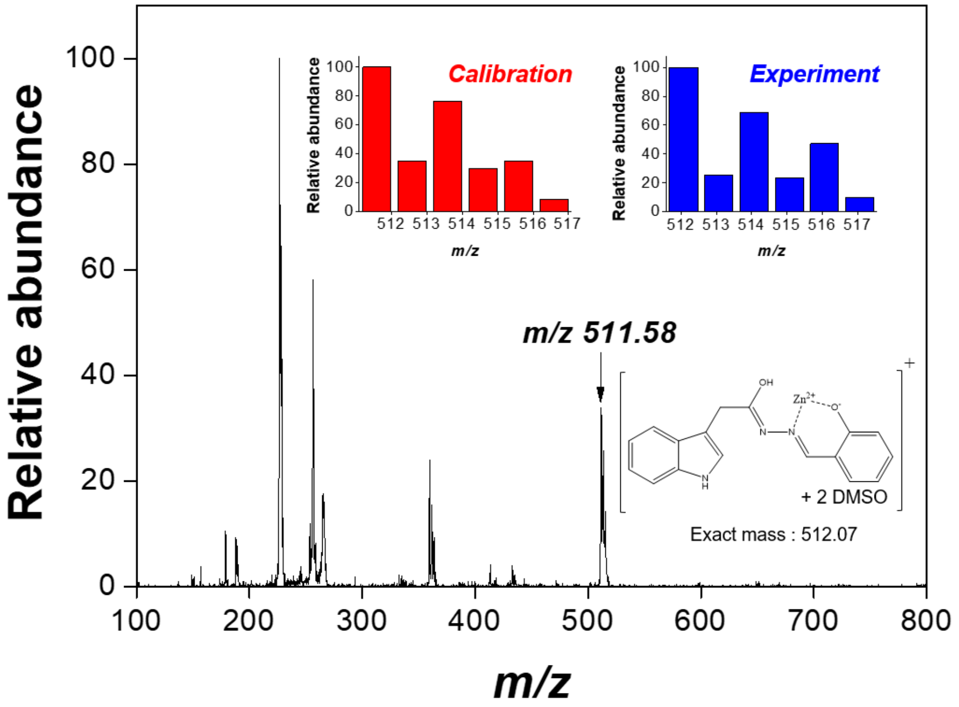

Figure S4. Positive-ion ESI mass spectrum of **IH-Sal** (100 μM) upon the addition of 1 equiv of Zn^{2+}.

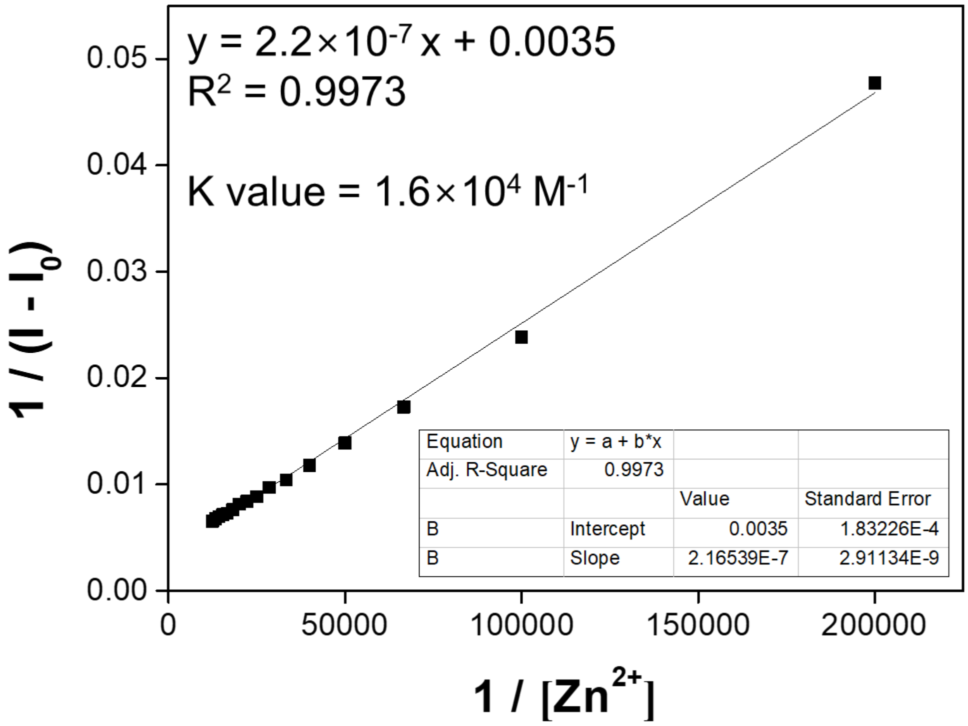

Figure S5. Benesi–Hildebrand equation plot (at 465 nm) of **IH-Sal** (10 μM) based on fluorescence titration, assuming 1:1 stoichiometry for association between **IH-Sal** and Zn^{2+}.

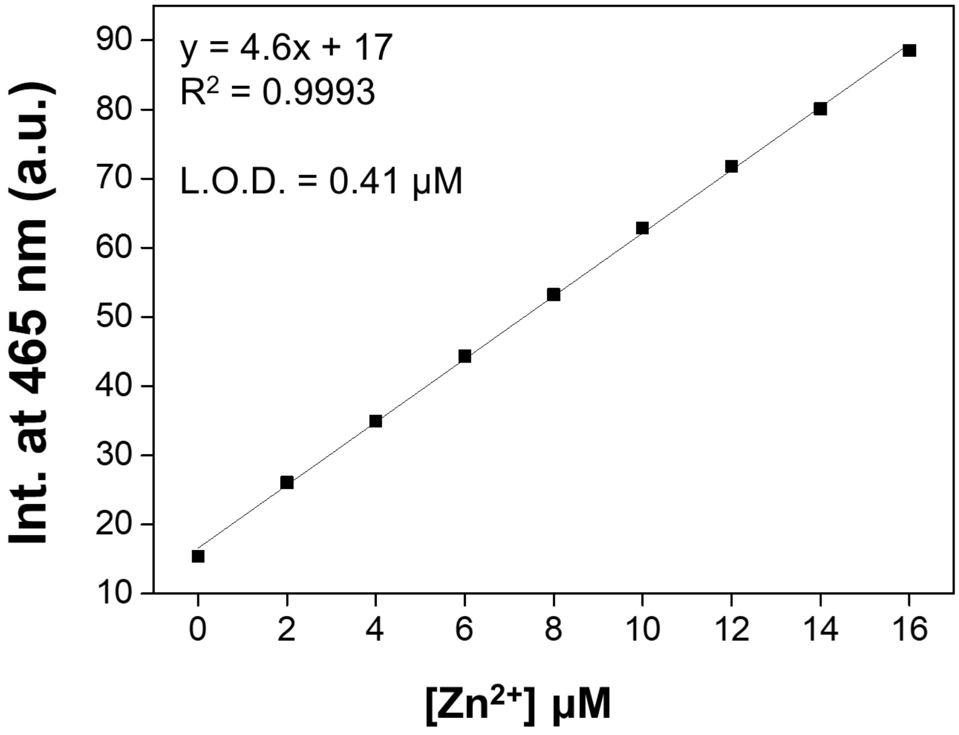

Figure S6. Calibration curve of **IH-Sal** as a function of Zn^{2+} concentration. [**IH-Sal**] = 10 μM and [Zn^{2+}] = 0.00–16.00 μM in bis-tris buffer solution (10 mM, pH 7.0, λ_{ex} = 369 nm).

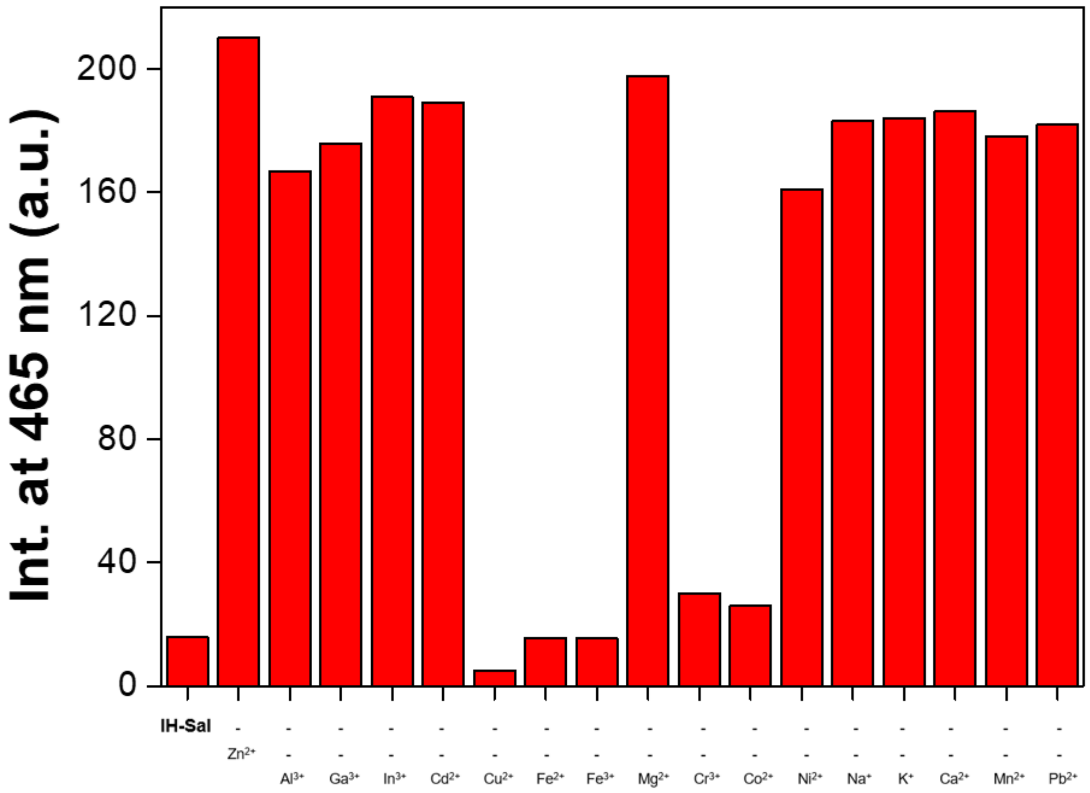

Figure S7. Competitive selectivity of **IH-Sal** (10 μM) toward Zn^{2+} (8.5 equiv) in the presence of other metal ions (8.5 equiv, λ_{ex} = 369 nm).

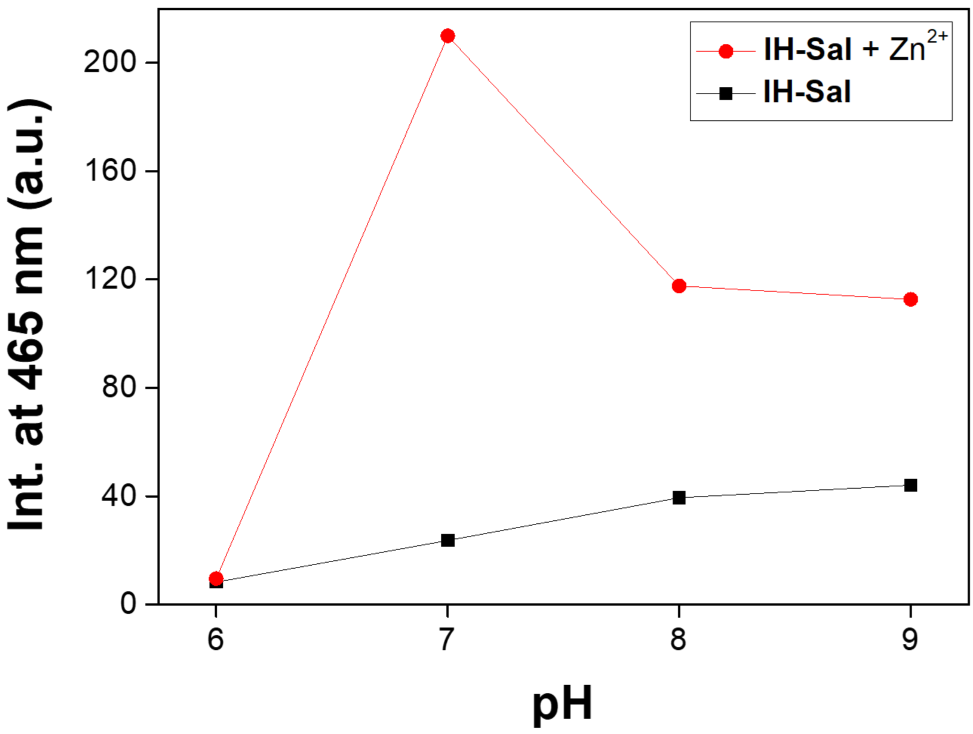

Figure S8. Fluorescent intensity of **IH-Sal** (10 μM) and **IH-Sal-**Zn^{2+} species, respectively, at different pH values (6–9).

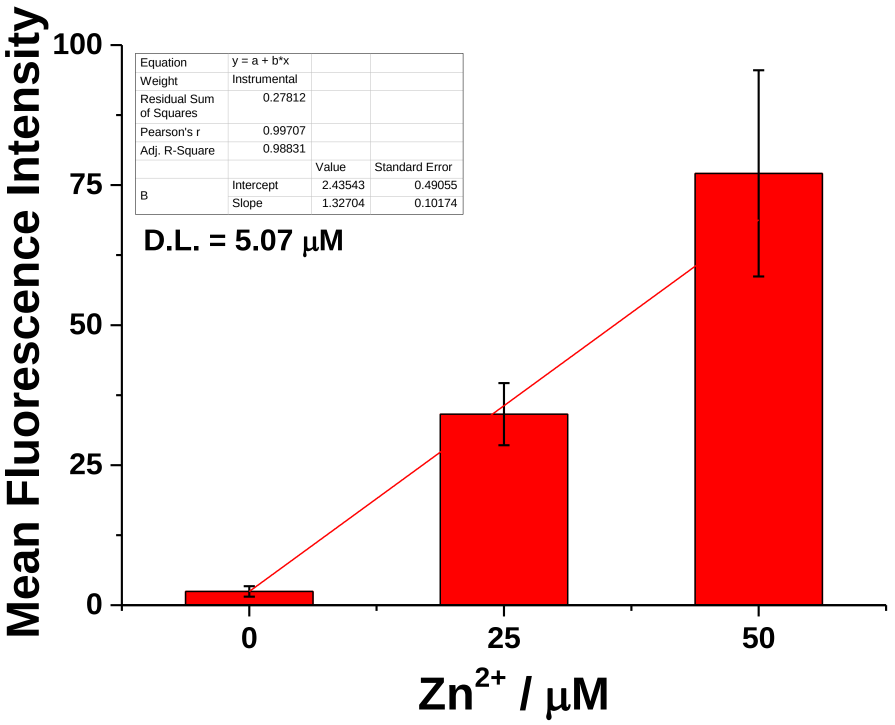

Figure S9. Quantification of mean fluorescence intensity in Figure 7 (a_2, b_2 and c_2).

(a)

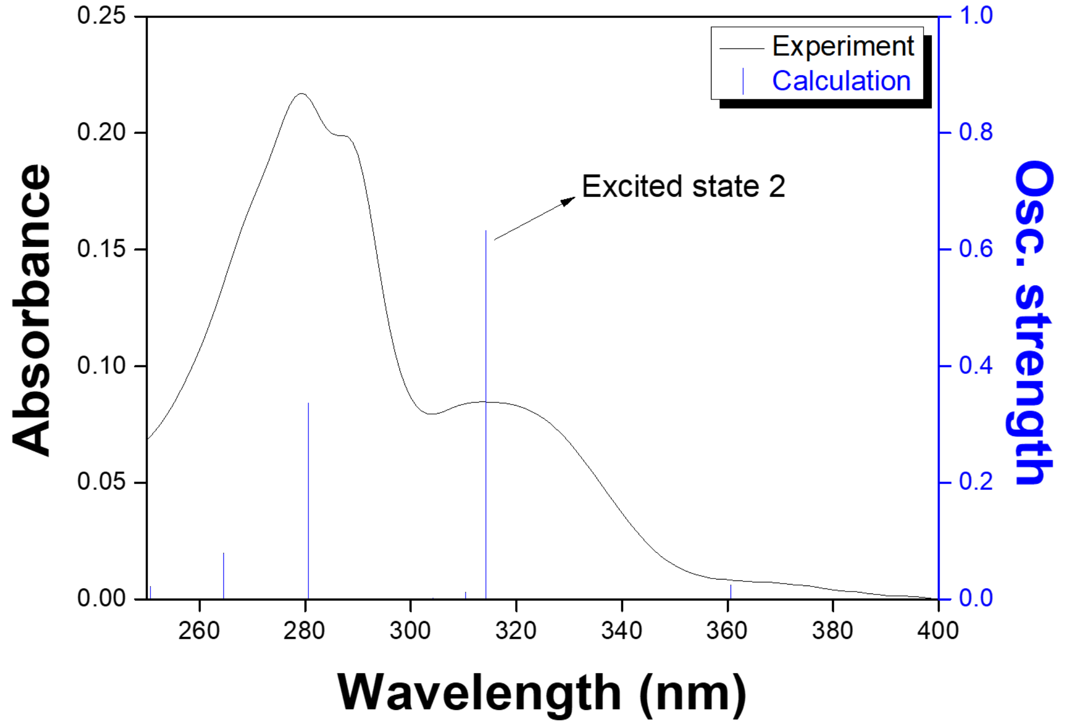

(b)

Excited state 2	Wavelength (nm)	Percent (%)	Main Character	Oscillator strength
HOMO-1 → LUMO	314.18	88%	π-π* transition	0.6326

Figure S10. (**a**) The theoretical excitation energies and the experimental UV–Vis spectrum of **IH-Sal**. (**b**) The major electronic transition energies and molecular orbital contributions of **IH-Sal**.

(a)

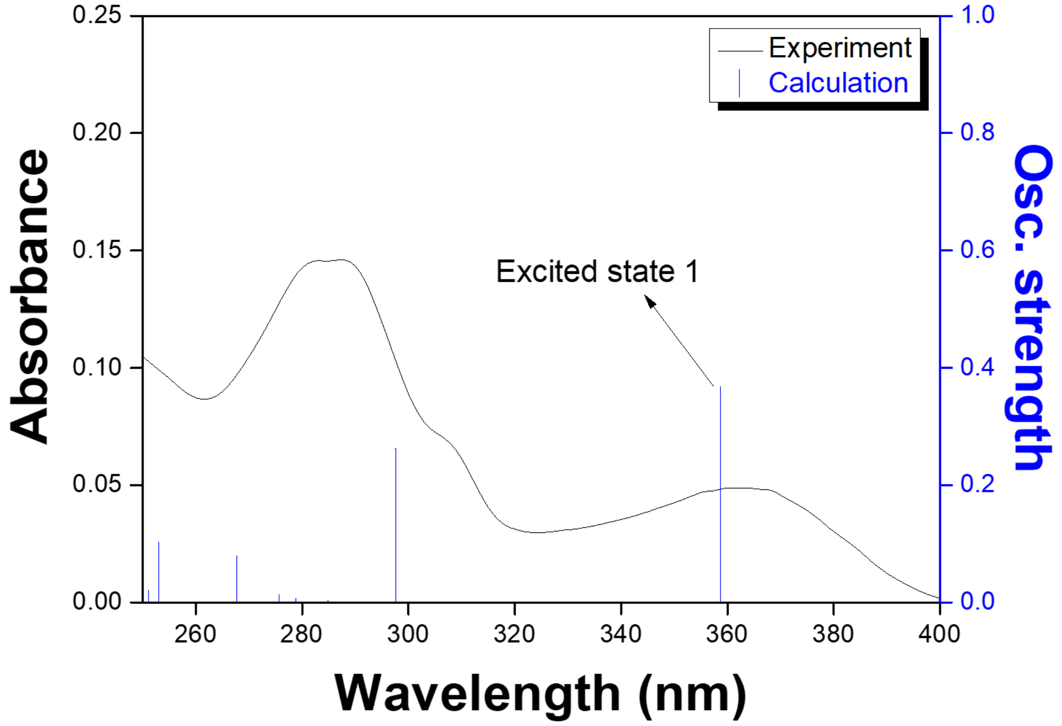

(b)

Excited state 1	Wavelength (nm)	Percent (%)	Main Character	Oscillator strength
HOMO → LUMO	358.71	97%	π-π* transition	0.3671

Figure S11. (**a**) The theoretical excitation energies and the experimental UV–Vis spectrum of **IH-Sal**-Zn^{2+}. (**b**) The major electronic transition energies and molecular orbital contributions of **IH-Sal**-Zn^{2+}.

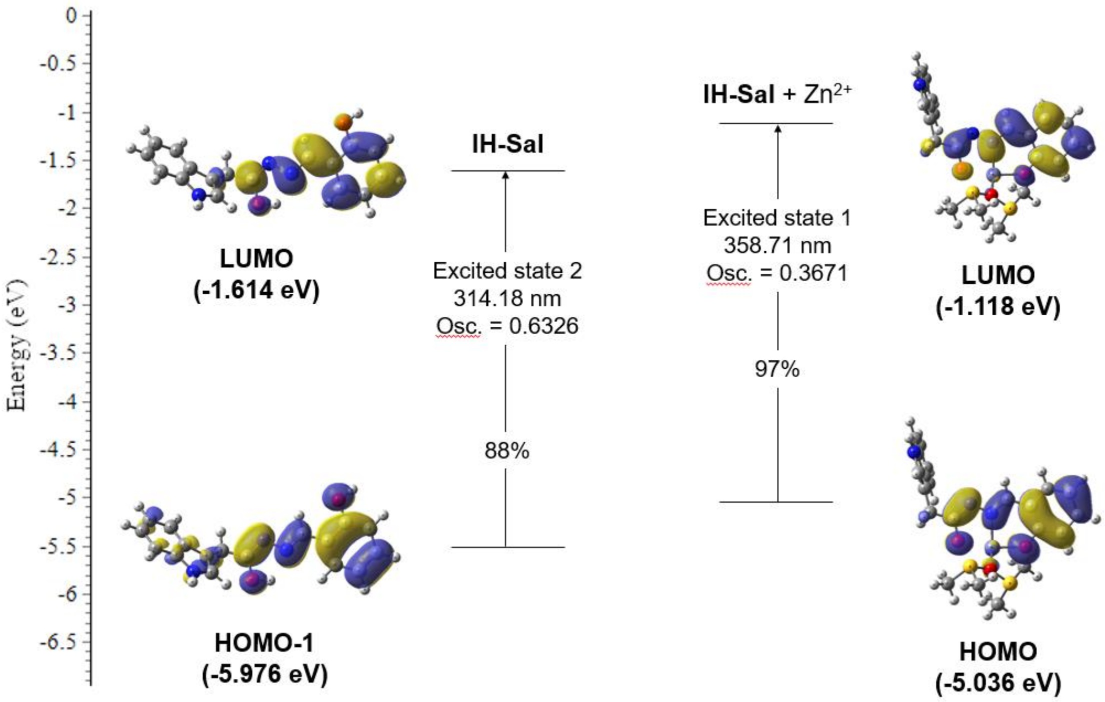

Figure S12. The major molecular orbital transitions and excitation energies of **IH-Sal** and **IH-Sal**-Zn^{2+}.

References

1. Xu, T.; Duan, H.; Wang, X.; Meng, X.; Bu, J. Fluorescence sensors for Zn^{2+} based on conjugated indole Schiff base. *Spectrochim. Acta Part A Mol. Biomol. Spectrosc.* **2015**, *138*, 596–602, doi:10.1016/j.saa.2014.11.098.
2. Dutta, K.; Deka, R.C.; Das, D.K. A new fluorescent and electrochemical Zn^{2+} ion sensor based on Schiff base derived from benzil and L-tryptophan. *Spectrochim. Acta Part A Mol. Biomol. Spectrosc.* **2014**, *124*, 124–129, doi:10.1016/j.saa.2013.12.090.
3. Taki, M.; Watanabe, Y.; Yamamoto, Y. Development of ratiometric fluorescent probe for zinc ion based on indole fluorophore. *Tetrahedron Lett.* **2009**, *50*, 1345–1347, doi:10.1016/j.tetlet.2009.01.026.
4. Singla, N.; Tripathi, A.; Rana, M.; Kishore Goswami, S.; Pathak, A.; Chowdhury, P. "Turn on/off" proton transfer based fluorescent sensor for selective detection of environmentally hazardous metal ions (Zn^{2+}, Pb^{2+}) in aqueous media. *J. Lumin.* **2015**, *165*, 46–55, doi:10.1016/j.jlumin.2015.04.007.
5. Li, L.; Dang, Y.Q.; Li, H.W.; Wang, B.; Wu, Y. Fluorescent chemosensor based on Schiff base for selective detection of zinc(II) in aqueous solution. *Tetrahedron Lett.* **2010**, *51*, 618–621, doi:10.1016/j.tetlet.2009.11.070.
